# METTL3/YTHDC1-medicated m6A modification of circRNA3634 regulates the proliferation and differentiation of antler chondrocytes by miR-124486-5-MAPK1 axis

**DOI:** 10.1186/s11658-023-00515-z

**Published:** 2023-12-07

**Authors:** Mengmeng Song, Haibo Yao, Zitong Sun, Danyang Chen, Xiwen Xu, Guohui Long, Lei Wu, Wei Hu

**Affiliations:** College of Life Science, Jilin Agriculture University, Changchun, 130118 China

**Keywords:** Antler chondrocyte, Cell proliferation, MAPK1, circRNA3634, m6A, YTHDC1

## Abstract

**Background:**

The deer antler, a remarkable mammalian appendage, has a growth rate surpassing that of any other known osseous organ. Emerging evidence indicates that circRNA and MAPK1 play critical roles in chondrocytes. Thus, exploration of their functions in antler chondrocytes will help us to understand the mechanism regulating the rapid antler growth.

**Methods:**

qRT-PCR, western blot, and immunohistochemistry were used to assess the expression of mRNAs and proteins. CCK-8, EdU, Cell migration, ALP activity detection, and ALP staining examined the effects of MAPK1 in antler chondrocytes. FISH, RIP, and luciferase assays were performed to evaluate the interactions among circRNA3634/MAPK1 and miR-124486-5. RIP and RAP assays proved the binding interaction between circRNA3634 and RBPs. Me-RIP was used to determine the m6A methylation modification of circRNA3634.

**Results:**

This study revealed high MAPK1 expression in antler cartilage tissue. Overexpression of MAPK1 promoted the proliferation, migration, and differentiation of antler chondrocytes and increased the expression of MAPK3, RAF1, MEK1, RUNX2, and SOX9. The silencing of MAPK1 had the opposite effect. CircRNA3634 was found to act as a molecular sponge for miR-124486-5, leading to increased MAPK1 expression and enhanced proliferation and migration of antler chondrocytes through competitive miR-124486-5 binding. We discovered that METTL3 mediates m6A modification near the splicing site of circRNA3634 and is involved in the proliferation and differentiation of antler chondrocytes. The m6A reader YTHDC1 facilitated the nuclear export of circRNA3634 in an m6A-dependent manner. Our results indicate that m6A-modified circRNA3634 promotes the proliferation of antler chondrocytes by targeting MAPK1 and show that the nuclear export of circRNA3634 is related to the expression of YTHDC1, suggesting that circRNA3634 could represent a critical regeneration marker for the antler.

**Conclusions:**

Our results revealed a novel m6A-modified circRNA3634 promoted the proliferation and differentiation of antler chondrocytes by regulating MAPK1. The nuclear export of circRNA3634 was related to the expression of YTHDC1.

## Introduction

Antlers are the unboned and velvety young horns of male deer. They are a bony organ that can be shed and completely regenerated from the pedicles annually [1]. The development of antlers begins in the antlerogenic periosteum, and the ossification processes involve intramembranous, transitional, and modified endochondral ossification [2]. The periodic regeneration and osteogenesis of antlers are complex biological processes regulated by various factors, including hormones, growth factors, light, temperature, feeding, and signaling pathways [3–5]. A recent study showed the MAPK, BMP, and TGF-β signaling pathways are involved in antler apical antler blastema progenitor cells (ABPCs) [6]. Our previous research focused on the TGF-β signaling system [7–9]. TGF-β signals are transmitted through Smad signaling and Smad-independent pathways, such as those of MAPK [10]. The RAF/MEK/ERK pathway was the first mammalian MAPK pathway to be described [11]. The activation of receptor tyrosine kinases by growth factors is the primary trigger of the ERK signaling pathway and leads to the activation of RAF, which phosphorylates serines of MEK. MEK continues to phosphorylate threonine and tyrosine of ERK1/2. Once activated, ERK1/2 translocates to the nucleus and triggers the activation of transcription factors, thereby regulating cell genesis and development [12]. ERKs participate in the regulation of chondrocyte proliferation, differentiation, and the expression of related genes [13–15]. Recent evidence also suggests the importance of the MAPK1 gene in the rapid growth of antlers [6, 16]. Thus, the investigation of the specific roles of MAPK1 in antler regeneration and development will contribute to unraveling the mysteries surrounding rapid antler growth.

CircRNAs are a class of non-coding RNAs characterized by closed-loop structures with no 3' and 5' polar ends that are expressed abundantly in eukaryotic cells [17]. They are involved in various physiological processes through different mechanisms. They act as miRNA and RNA binding protein (RBP) "sponges" [18–20] and participate in translation into proteins [21], and the regulation of transcription [22, 23]. For example, circASAP1 promotes the proliferation and invasion of HCC cells by modulating the miR-326/miR-532-5p-MAPK1 axis [24]. Similarly, circ-0001679 maintained high MAPK1 expression by suppressing miR-338-3p [25]. CircRNAs are more stable than linear RNAs [26]. The investigation of their role in antler chondrocytes and the underlying mechanisms will enhance our understanding of antler apical tissue growth and development.

N6-methyladenosine (m6A) modification, an emerging topic in epigenetic regulation, is essential for several biological activities and characteristics, including RNA splicing, export, translation, and stability [27–29]. Methylation transferases (writers), demethylases (erasers), and m6A-binding proteins (readers) work in coordination [30]. METTL3 is the first identified m6A methyltransferase with a catalytic function, and its action can be reversed by the m6A demethylases FTO and ALKBH5 [31]. Readers of m6A modifications selectively recognize m6A sequences and bind to m6A motifs, thereby influencing RNA function [32]. Among them, YTHDC1 is the sole m6A-binding protein localized in the nucleus; it participates in RNA transcription, splicing, nuclear export, and degradation [28, 33–35]. The involvement of m6A modification in the proliferation and differentiation of antler chondrocytes has not been reported, and is an important area for further investigation.

The objectives of this study were to investigate the regulation of the MAPK signaling pathway by circRNA3634 and its impact on the proliferation and differentiation of sika deer (*Cervus nippon*) antler chondrocytes from the perspective of RNomics. This report is the first to describe the molecular mechanism of RNA epigenetic modification that regulates the proliferation and differentiation of antler chondrocytes. The findings of this study provide new insights into the regulatory mechanisms underlying rapid antler regeneration and development.

## Materials and methods

### Antler chondrocytes isolation and culture

Mesenchyme and cartilage tissues from *C nippon* antlers were isolated, fragmented, and enzymatically digested using collagenase I, collagenase II, and hyaluronidase (Sigma) under sterile conditions. The obtained antler cells were cultured in DMEM (Gibco) supplemented with 15% FBS (Gibco) at 37 °C in a 5% CO_2_ incubator.

### Total RNA extraction and qRT-PCR

Total RNA was extracted using TRIzol reagent (TransGen Biotech). For the quantitative detection of mRNAs and circRNAs, a first-strand cDNA synthesis kit with gDNA removal and SYBR Green reagent (TransGen Biotech) was used. Reverse transcription and quantification of miRNAs were performed using the miDETECT A Track Kit (RiboBio). The mRNA primers were synthesized by Sangon Biotech and the miRNA primers were obtained from RiboBio. The relative expression of mRNAs and miRNA was analyzed using the 2^−∆∆Ct^ method with β-actin and U6, respectively, serving as internal reference genes. The primer sequences are provided in Table 1.

**Table 1:** Primers for qRT-PCR

Name	Sense	Antisense
MAPK1	AACCTTCCAACCTGCTGCTC	CGTACTCTGTCAAGAACCCTGTG
MAPK3	GATCTGTGATTTCGGTCTTGC	ATGGACTTGGTGTAGCCCTTG
RAF1	CTGTTGGTGATGGTGGGGTC	GGGCTGGAGGTGTTGAATGT
MEK1	ACTCCAGGGGACCCATTACTC	CGACCGCCATCTCAACCA
RUNX2	AGCTATTAAAGTCACAGTGGATGG	GGCGATCAGAGAACAAACTAGG
SOX9	CCCCCCACTACCCCCAAAA	CGCTACTCAGTTCGCCGATG
circRNA987	GTGAATCTTTAGAGCCTGTGG	GCCTGCTGGTTACTCATCTT
circRNA517	TCTCAGTGTTGCCCTGTTC	GGCTTTGGTGGTTCTGTAGT
circRNA3634 divergent primer	CGTAGACCGCCACCTGAA	TGCTGATGGACTCTGATGCT
circRNA3634 convergent primer	CAAGGACTATCACAGGAAGCC	CCAAATATGCAGCAAGGTCT
COG3	GAAACTGTGTGTGTCGTTGTATGA	CGTCTGAATGTAGATGTGGGTC
METTL3	GCACTGTCTCCAACCTTCCGT	GGTGGTGTAGCAACTTCTTCTCTAAT
YTHDC1	TTCAGGAGTTCGCCGAGATGTA	TAAGGATGGTGGGGAGGTTGTT
β-actin	GTCCGTGACATCAAGGAGAAGC	AAGGTAGTTTCGTGAATGCCGC

### Plasmid construction, siRNA, miRNA mimic, and miRNA inhibitor

The PCR product of the *C*. *nippon* MAPK1 gene was inserted between the XbaI and HindIII enzyme sites to construct the pcDNA3.1-MAPK1 overexpression plasmid. The construction of the pcDNA3.1-circRNA3634 overexpression plasmid and the synthesis of an siRNA targeting circRNA3634 and a negative control were completed by Hanbio. Wild-type and mutant dual luciferase reporter plasmids containing partial 3'UTR sequences of MAPK1 and circRNA3634 were constructed by Comate Bioscience. siRNAs against MAPK1, METTL3, YTHDC1, and the miR-124486-5 mimic and inhibitor were purchased from RiboBio. The 5'FAM-si-MAPK1 was purchased from Genepharma.

### Cell transfection

According to the manufacturer's instructions, overexpression plasmids, siRNAs, and the miRNA mimic and inhibitor were transfected into antler chondrocytes using Lipo 8000 transfection reagent (Beyotime).

### RNase R and actinomycin D treatments

For the RNase R treatment, 10 U RNase R (Geneseed) was added to 5 μg total RNA from antler cartilage tissue, and incubated at 37 °C for 30 min. The expression of mRNAs and circRNAs was then detected by qRT-PCR. Antler chondrocytes were also treated with 2 μg/mL Act D (Selleck). Cells were collected at predetermined intervals, and qRT-PCR was used to identify the expression of linear mRNAs and circRNAs.

### Cell proliferation assay

Cell proliferation was detected using CCK-8 and EdU assays. For the CCK-8 assay, antler chondrocytes were seeded in 96-well plates and transfected with the required reagents using Lipo 8000. They were incubated with 100 μL 10% CCK-8 solution (Bioss) at 37 °C for 2 h. Absorbance at 450 nm was measured to determine cell viability. For the EdU assay, antler chondrocytes were seeded in 12-well plates and transfected for 48 h. They were then incubated with 10 μM EdU at 37 °C for 4 h, according to the manufacturer’s instructions (Beyotime). After fixation with 4% paraformaldehyde, the click reaction solution was added, and the nuclei were stained with DAPI. Images were captured using a fluorescence microscope (AX10 Carl Zeiss Jena).

### Cell migration assay

Antler chondrocytes were seeded in a 12-well plate and streaked vertically with 200 μL pipette tips. The cells were washed with PBS and cultured in DMEM supplemented with 1% FBS. After transfection with the desired reagents, the antler chondrocytes were photographed and sampled at 0, 12, 24, and 36 h for further analysis.

### ALP activity measurement and ALP staining

ALP activity in antler chondrocytes was assessed using an ALP activity assay kit (Beyotime). Total protein was extracted from the cells, and a chromogenic substrate was added. After the termination of the resulting reaction, the OD value of each sample was measured at 405 nm, and a relative ALP activity value was calculated. For ALP staining, the BCIP/NBT ALP staining detection kit (Beyotime) was used and images were captured.

### Immunohistochemistry

Fresh antler mesenchyme and cartilage tissues were collected, fixed, sectioned, and embedded in paraffin. After overnight incubation with an antibody against ERK1/2 (1:300, CST) at 4 °C. The sections were incubated with an anti-rabbit horseradish peroxidase (HRP) antibody (1:200, Bioss) at room temperature for 30 min. The expression of the ERK1/2 protein was detected using DAB chromogenic reagent (Beyotime). Images were captured under a microscope (Leica).

### Immunofluorescence

Antler chondrocytes were fixed, permeabilized, blocked, and incubated with an ERK1/2 antibody (1:800, CST), followed by FITC-conjugated secondary antibodies (1:200, Bioss). The nuclei were stained with DAPI (1:1000, Bioss), and images were captured under a fluorescence microscope (AX10; Carl Zeiss Jena).

### Western blot

Total protein was extracted from antler chondrocytes using RIPA lysis buffer (Beyotime). SDS-PAGE was used to separate the protein, and PVDF membranes (Millipore) were used to transfer it. The membranes were blocked with 5% skim milk (BD) and incubated with antibodies against p-ERK1/2 (1:1000, Bioss), t-ERK1/2 (1:2000, CST), RAF1 (1:1000, Abcam), MEK1 (1:10,000, Abcam), RUNX2 (1:2000, Abcam), SOX9 (1:2000, CST), METTL3 (1:2000, Proteintech), YTHDC1 (1:2000, Proteintech), and β-actin (1:2000, OriGene). After washing with TBST, the membranes were incubated with anti-mouse or anti-rabbit HRP-conjugated secondary antibodies (1:2000, Bioss). The signals were detected using a chemiluminescence kit (Millipore), with β-actin serving as the control.

### RNA fluorescence in situ hybridization (FISH)

The subcellular localization of circRNA3634 was detected by FISH (Genepharma). A Cy3-labeled circRNA3634 probe was provided by Genepharma, and the nuclei were stained with DAPI. Images were captured under a fluorescence microscope (AX10; Carl Zeiss Jena).

### Luciferase reporter assay

Antler chondrocytes were seeded in 24-well plates and co-transfected with the NC or miR-124486-5 mimic, along with the dual luciferase reporter plasmid using Lipo 8000 (Beyotime). After 48 h, cells were collected and lysed with lysate. Luciferase activity was measured using a dual luciferase assay kit (YEASEN Biotech) following the manufacturer's instructions.

### RNA immunoprecipitation (RIP)

RIP was performed using a commercially available kit (Bersin Bio). Antler chondrocytes were lysed with RIP lysis buffer. The lysates were then incubated with antibodies against AGO2, METTL3, YTHDC1, and IgG. Then, protein A/G beads were added to the lysates, followed by incubation for 1 h at 4 °C. The beads were then washed to remove non-specifically bound proteins. RNA was extracted from the beads and subjected to qRT-PCR for the determination of the circRNA3634 or miR-124486-5 enrichment caused by each antibody.

### Methylated RNA immunoprecipitation (MeRIP)

For the quantitative analysis of m6A-modified circRNA3634, MeRIP-PCR was performed. 10 μg total RNA was disrupted into fragments about 300 nt in size. The fragments were incubated with 5 μg m6A antibody (Proteintech) or IgG antibody at 4 °C for 4 h. Then, protein A/G beads were added to the mixture followed by incubation for 1 h at 4 °C. Elution buffer was used to elute the RNA-bead complexes. qRT-PCR was used to determine circRNA3634 enrichment.

### RNA antisense purification (RAP)

According to the manufacturer's instructions, the RAP kit (Bersin Bio) was used for RAP determination. About 4 × 10^7^ antler chondrocytes were collected and cross-linked with PBS containing formaldehyde for 10 min to preserve RNA–protein interactions. The cross-linked cells were lysed with cell lysis buffer. 5 μL biotin-labeled probe was added to the RAP sample, hybridized at 37 °C for 30 min, incubated at 50 °C for 5 min, and hybridized at 37 °C for 180 min. Finally, streptavidin beads were incubated with RAP samples at room temperature for 30 min, and the proteins on the beads were eluted. The expression of proteins bound to circRNA3634 was analyzed by mass spectrometry.

### Statistical analysis

The experimental data are presented as means ± standard deviations. The statistical analysis was performed using GraphPad Prism v8.3. Data were compared between groups using paired or unpaired t-tests. The significance level was set to *p* < 0.05.

## Results

### MAPK1 promotes the proliferation, migration, and differentiation of antler chondrocytes

**Fig. 1:**
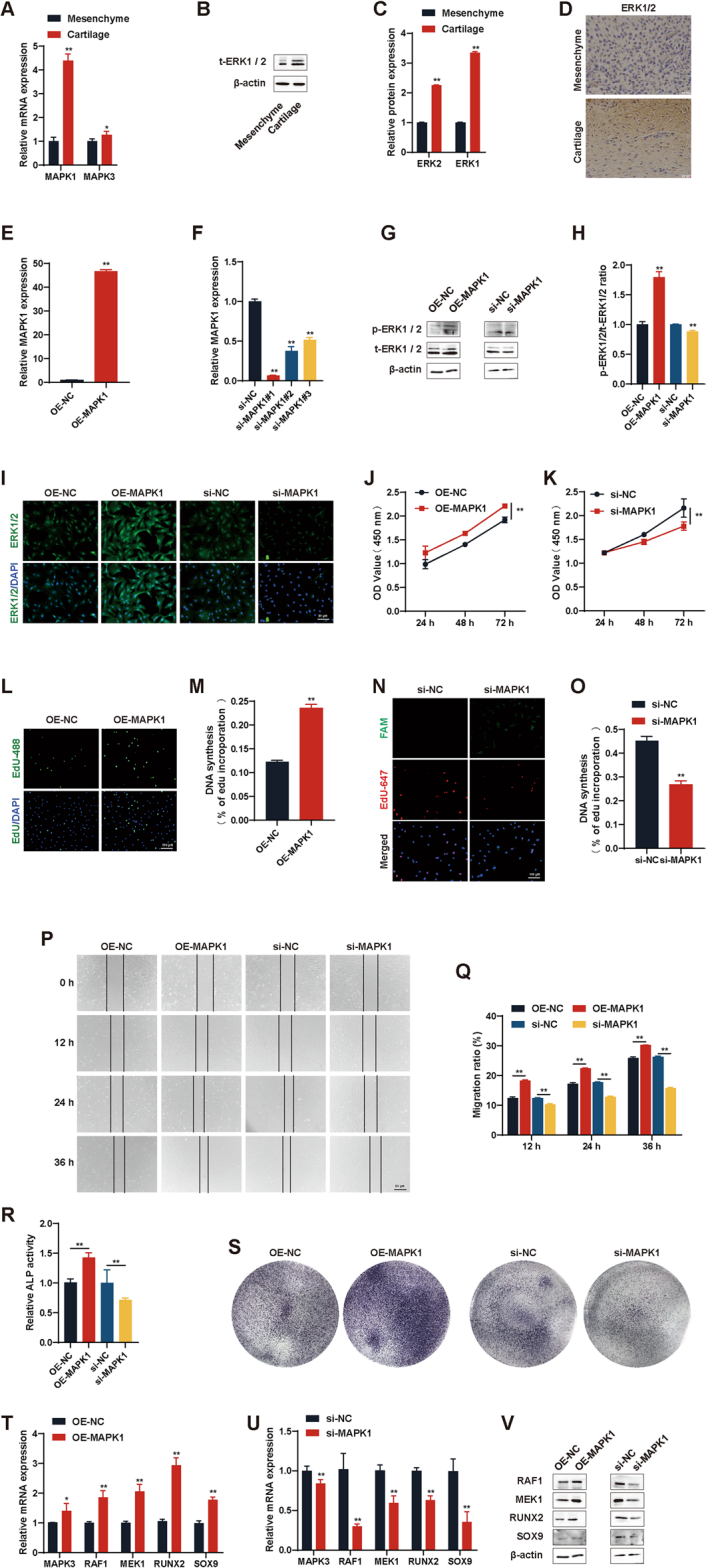
MAPK1 promotes the proliferation, migration, and differentiation of antler chondrocytes. **A**–**D** qRT-PCR, western blot, and immunohistochemical analyses showed high expression of MAPK1 and MAPK3 in antler cartilage tissue. Scale bar, 25 μm. **E** and **F** Antler chondrocytes were transfected with the pcDNA3.1-MAPK1 plasmid or MAPK1 siRNAs along with negative controls. qRT-PCR was performed to detect MAPK1 expression. **G–I** Western blots and immunofluorescence images showing increased ERK1/2 expression with OE-MAPK1 and decreased expression with si-MAPK1. Scale bar, 50 μm. **J–O** CCK-8 and EdU results showing that OE-MAPK1 promoted cell proliferation and si-MAPK1 inhibited it. Scale bar, 100 μm. **P** and **Q** A cell migration assay showed that OE-MAPK1 enhanced cell migration and si-MAPK1 inhibited it. Scale bar, 50 μm. **R** OE-MAPK1 increased ALP activity and si-MAPK1 decreased it. **S** OE-MAPK1 intensified ALP staining and si-MAPK1 attenuated it. **T** and **U** qRT-PCR showed that OE-MAPK1 upregulated the mRNA expression of MAPK3, RAF1, MEK1, RUNX2, and SOX9 and si-MAPK1 down-regulated it. **V** Western blots of RAF1, MEK1, RUNX2, and SOX9 protein expression in antler chondrocytes. *p < 0.05, **p < 0.01. N.S., no signification

qRT-PCR, western blot, and immunohistochemical analyses revealed high expression of the MAPK1 and MAPK3 gene in *C. nippon* antler cartilage tissues (Fig. 1A–D). To investigate the biological function of MAPK1, we constructed the pcDNA3.1-MAPK1 plasmid and transfected it into antler chondrocytes to overexpress MAPK1(OE-MAPK1) (Fig. 1E, G, I). Additionally, three siRNAs targeting MAPK1 were designed and used to silence MAPK1 expression in antler chondrocytes, as confirmed by qRT-PCR, western blot, and immunofluorescence analyses (Fig. 1F, G, I). Moreover, we found that OE-MAPK1 increased the ratio of pERK1/2/t-ERK1/2, while si-MAPK1 had the opposite effect (Fig. 1H).

CCK-8 assays (Fig. 1J–K) and EdU staining (Fig. 1L–O) demonstrated that OE-MAPK1 significantly promoted cell proliferation, and silencing MAPK1 (si-MAPK1) inhibited it. A cell migration assay revealed that OE-MAPK1 enhanced cell migration ability and si-MAPK1 significantly impaired it (Fig. 1P-Q).

Alkaline phosphatase (ALP), a byproduct of osteoblast activity, has been utilized widely as a marker of early bone tissue formation. Its upregulation serves as an indicator of osteoblast differentiation [36]. Additionally, RUNX2 and SOX9 are widely recognized as crucial transcriptional regulatory factors involved in chondrogenic differentiation [37, 38]. An ALP activity assay and ALP staining showed that OE-MAPK1 increased ALP activity and osteogenic differentiation in antler chondrocytes, and that si-MAPK1 had the opposite effect (Fig. 1R-S). MAPK3, also known as ERK1, is a component of the MAPK/ERK signaling pathway. RAF1 and MEK1 are upstream kinases of ERK1/2. qRT-PCR and western blot indicated that OE-MAPK1 upregulated the expression of MAPK3 (ERK1), RAF1, MEK1, RUNX2, and SOX9 at the mRNA and protein levels; si-MAPK1 resulted in the down-regulation of this expression (Fig. 1T–V).

#### The characteristics of circRNA3634 in antler chondrocytes

**Fig. 2:**
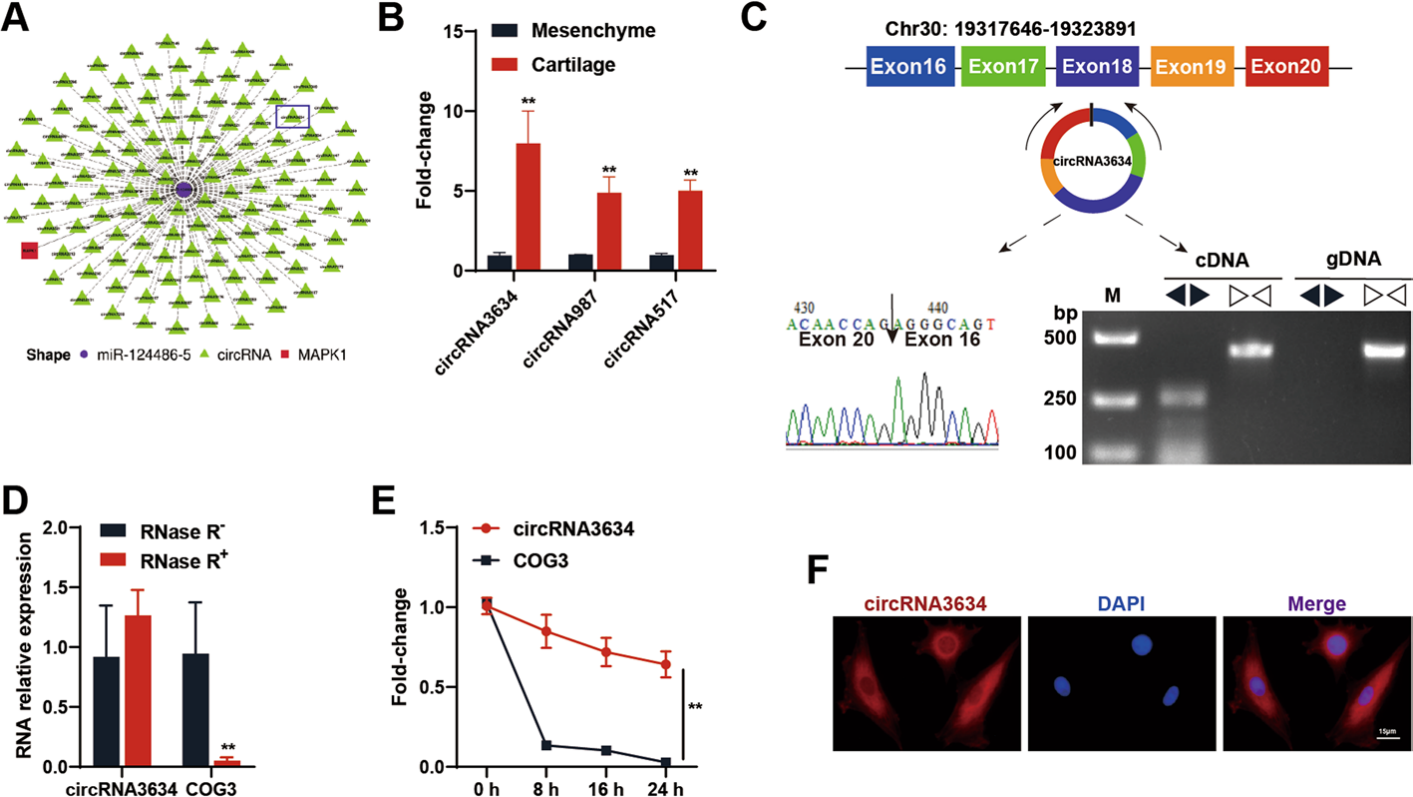
Identification of circRNA3634 in antler chondrocytes. **A** Prediction of the circRNA-miR-124486-5-MAPK1 axis. **B** qRT-PCR was performed to measure the expression of circRNAs in mesenchyme and cartilage tissues. **C** Schematic diagram illustrating that circRNA3634 is formed through the back-splicing of COG3 pre-mRNA. Sanger sequencing and agarose gel electrophoresis confirmed the back-splicing site. **D** Expression of circRNA3634 and COG3 mRNA assessed after treatment with RNase R. **E** Levels of circRNA3634 and COG3 mRNA determined by qRT-PCR following Act D treatment. **F** FISH experiments were conducted to detect circRNA3634 in antler chondrocytes. Nuclei were stained with DAPI (blue) and circRNA3634 probes were labeled with Cy3 (red). Scale bar, 15 μm. *p < 0.05, **p < 0.01. N.S., no signification

Following previous work [39], we used bioinformatics tools (TargetScan and miRanda) to predict the circRNA-miRNA-MAPK1 axis. Along this axis, miR-124486-5 binds to the 3’ UTR of MAPK1. Additionally, we found 124 circRNAs that bind to miR-124486-5 (Fig. 2A). Based on the whole transcriptome sequencing results, we selected three circRNAs with high expression and detected their differential expression in antler cartilage and mesenchyme tissues. Among them, circRNA3634 showed the most significant difference in expression between tissues and was selected for further investigation (Fig. 2B). The genomic location of circRNA3634 was determined to be at CM008037.1: 19317646–19323891. It is formed by the covalent closed circular RNA connecting the 3'-end G base with the 5'-end A base of the COG3 pre-mRNA, with a length of 530 nt (Fig. 2C). The circular structure of circRNA3634 was confirmed by Sanger sequencing, which validated the back-splicing site (Fig. 2C). To verify its circular structure, we performed PCR using divergent and convergent primer. The divergent primer amplified circRNA3634 only from cDNA and the convergent primer amplified that from cDNA and gDNA (Fig. 2C). qRT-PCR assay performed after the treatment of antler chondrocyte total RNA with RNase R revealed that circRNA3634 was more stable than its linear COG3 mRNA (Fig. 2D). Treatment with actinomycin D (Act D) showed that the half-life of circRNA3634 was significantly longer than that of linear COG3 mRNA (Fig. 2E). Fluorescence in-situ hybridization (FISH) showed that circRNA3634 was localized mainly in the cytoplasm (Fig. 2F), suggesting that it may functions as a competing endogenous RNA (ceRNA).

#### CircRNA3634 promotes the proliferation and migration of antler chondrocytes

**Fig. 3:**
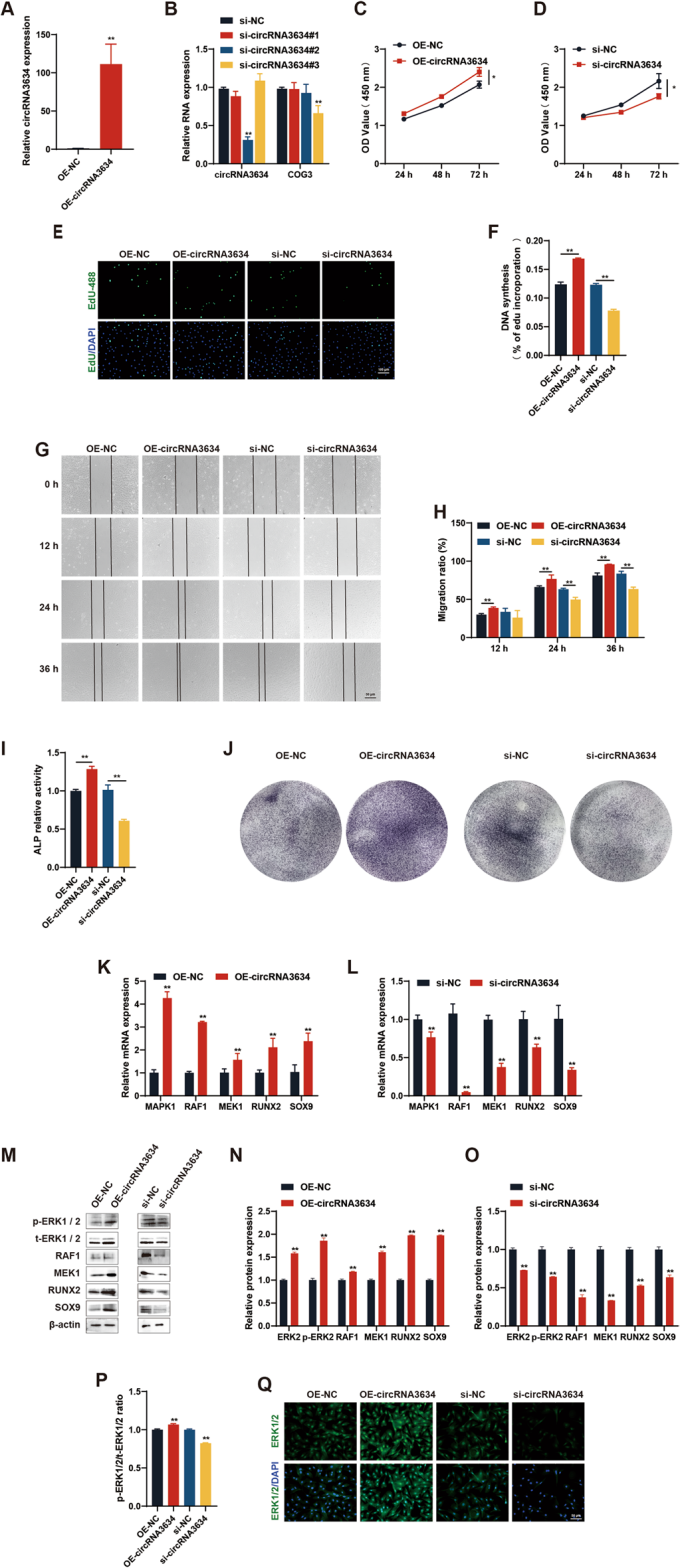
CircRNA3634 promotes the proliferation and differentiation of antler chondrocytes. **A** qRT-PCR revealed significant upregulation of circRNA3634 in antler chondrocytes following transfection with pcDNA3.1-circRNA3634. **B** Relative expression of circRNA3634 and COG3 mRNA after transfection of NC or circRNA3634 siRNA into antler chondrocytes, determined by qRT-PCR. **C–F** CCK-8 and EdU assays showed that OE-circRNA3634 promotes the proliferation of antler chondrocytes and si-circRNA3634 inhibits it. Scale bar, 100 μm. **G** and **H** A cell migration assay indicated that OE-circRNA3634 stimulated antler chondrocytes migration and si-circRNA3634 inhibited it. Scale bar, 50 μm. **I** OE-circRNA3634 increased ALP activity and si-circRNA3634 decreased it. **J** OE-circRNA3634 intensified ALP staining and si-circRNA3634 attenuated it. **K** and **L** Relative mRNA expression of MAPK1, RAF1, MEK1, RUNX2, and SOX9 in antler chondrocytes transfected with pcDNA3.1-circRNA3634 or circRNA3634 siRNA, determined by qRT-PCR. **M**–**P** Western blots of the protein expression of p-ERK1/2, t-ERK1/2, RAF1, MEK1, RUNX2, and SOX9 in antler chondrocytes transfected with pcDNA3.1-circRNA3634 or circRNA3634 siRNA. **Q** Immunofluorescence image showing the expression of t-ERK1/2 in antler chondrocytes transfected with pcDNA3.1-circRNA3634 or circRNA3634 siRNA. Scale bar, 50 μm. *p < 0.05, **p < 0.01. N.S., no signification

pcDNA3.1-circRNA3634 and its control plasmid were transfected into antler chondrocytes to study the biological function of circRNA3634. qRT-PCR revealed a significant increase in the expression of circRNA3634 in antler chondrocytes (Fig. 3A). Furthermore, we designed three siRNAs targeting the back-splicing site of circRNA3634 to interfere with endogenous expression. Among them, si-circRNA3634#2 effectively reduced circRNA3634 expression without affecting the COG3 mRNA level (Fig. 3B). Thus, si-circRNA3634#2 was used in a circRNA3634 functional loss analysis.

CCK-8 and EdU assays revealed that OE-circRNA3634 promoted the proliferation of antler chondrocytes and that si-circRNA3634 significantly inhibited it (Fig. 3C–F). A cell migration assay demonstrated that OE-circRNA3634 enhanced cell migration and si-circRNA3634 significantly suppressed it (Fig. 3G, H). ALP activity assay and ALP staining showed that OE-circRNA3634 increased ALP activity and osteogenic differentiation in antler chondrocytes, and that si-circRNA3634 had the opposite effect (FIg. 3I, J).

We investigated whether the expression of MAPK1 is influenced by circRNA3634. The results demonstrated that OE-circRNA3634 in antler chondrocytes led to significant increases in MAPK1 mRNA and t-ERK1/2 protein expression and si-circRNA3634 had the opposite effect (Fig. 3K–Q). We detected the expression of p-ERK1/2. The results showed that OE-circRNA3634 increased the ratio of p-ERK1/2, while si-circRNA3634 decreased it (Fig. 3M, P). It indicates that circRNA3634 is involved in the activation of the ERK signaling pathway. We examined the expression of the upstream kinases RAF1 and MEK1, as well as the downstream transcription factors RUNX2 and SOX9, which are involved in chondrocyte differentiation. OE-circRNA3634 led to increases in the mRNA and protein expression of RAF1, MEK1, RUNX2, and SOX9, and si-circRNA3634 led to decreases therein (Fig. 3K–O).

#### CircRNA3634 acts as a “molecular sponge” for miR-124486-5

**Fig. 4:**
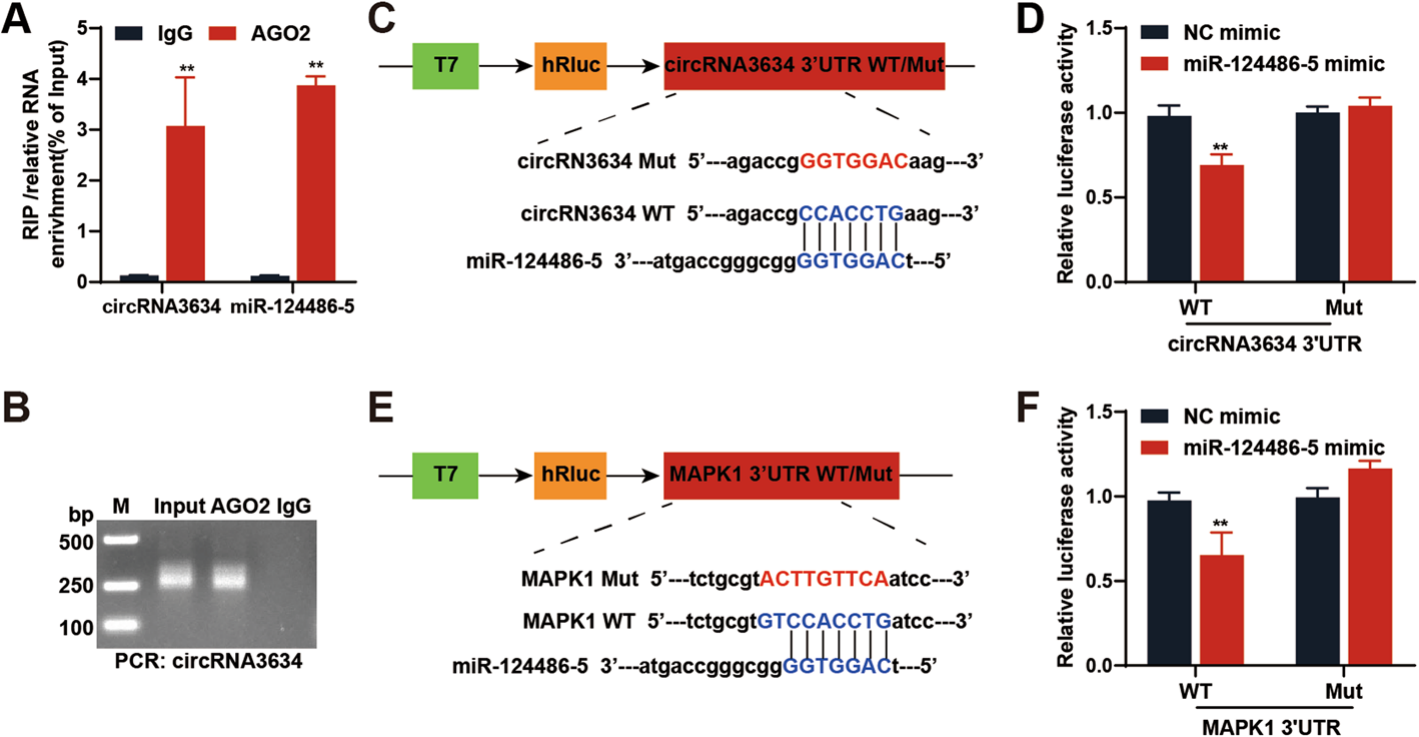
CircRNA3634 serves as a miR-124486-5 “sponge” in antler chondrocytes. **A** and **B** An RIP-PCR assay showed significant enrichment of circRNA3634 and miR-124486-5 in the AGO2 antibody immunoprecipitated complex. **C** Schematic diagram of the wild-type/mutant circRNA3634 dual-luciferase reporter vector. **D** Relative luciferase activity in antler chondrocytes co-transfected with the wild-type/mutant circRNA3634 dual-luciferase reporter vector and NC/miR-124486-5 mimic for 48 h. **E** Schematic diagram of the wild-type/mutant MAPK1 dual-luciferase reporter vector. **F** Relative luciferase activity in antler chondrocytes co-transfected with the wild-type/mutant MAPK1 dual-luciferase reporter vector and NC/miR-124486-5 mimic for 48 h. *p < 0.05, **p < 0.01. N.S., no signification

An RNA immunoprecipitation (RIP) assay showed significant enrichment of circRNA3634 and miR-124486-5 in the AGO2 antibody precipitated complex relative to the IgG antibody control (Fig. 4A, B), suggesting that circRNA3634 binds to miR-124486-5. A dual-luciferase assay demonstrated that the relative luciferase activity of wild-type circRNA3634 was reduced by the miR-124486-5 mimic, whereas that of mutant circRNA3634 did not change significantly, suggesting that circRNA3634 specifically targets and binds to miR-124486-5 (Fig. 4C, D). The miR-124486-5 mimic reduced the relative luciferase activity of wild-type MAPK1, but had no significant effect on that of mutant MAPK1, indicating that miR-124486-5 targets and binds to the 3ʹ UTR of MAPK1 (Fig. 4E, F).

#### MiR-124486-5 inhibits the proliferation and migration of antler chondrocytes

**Fig. 5:**
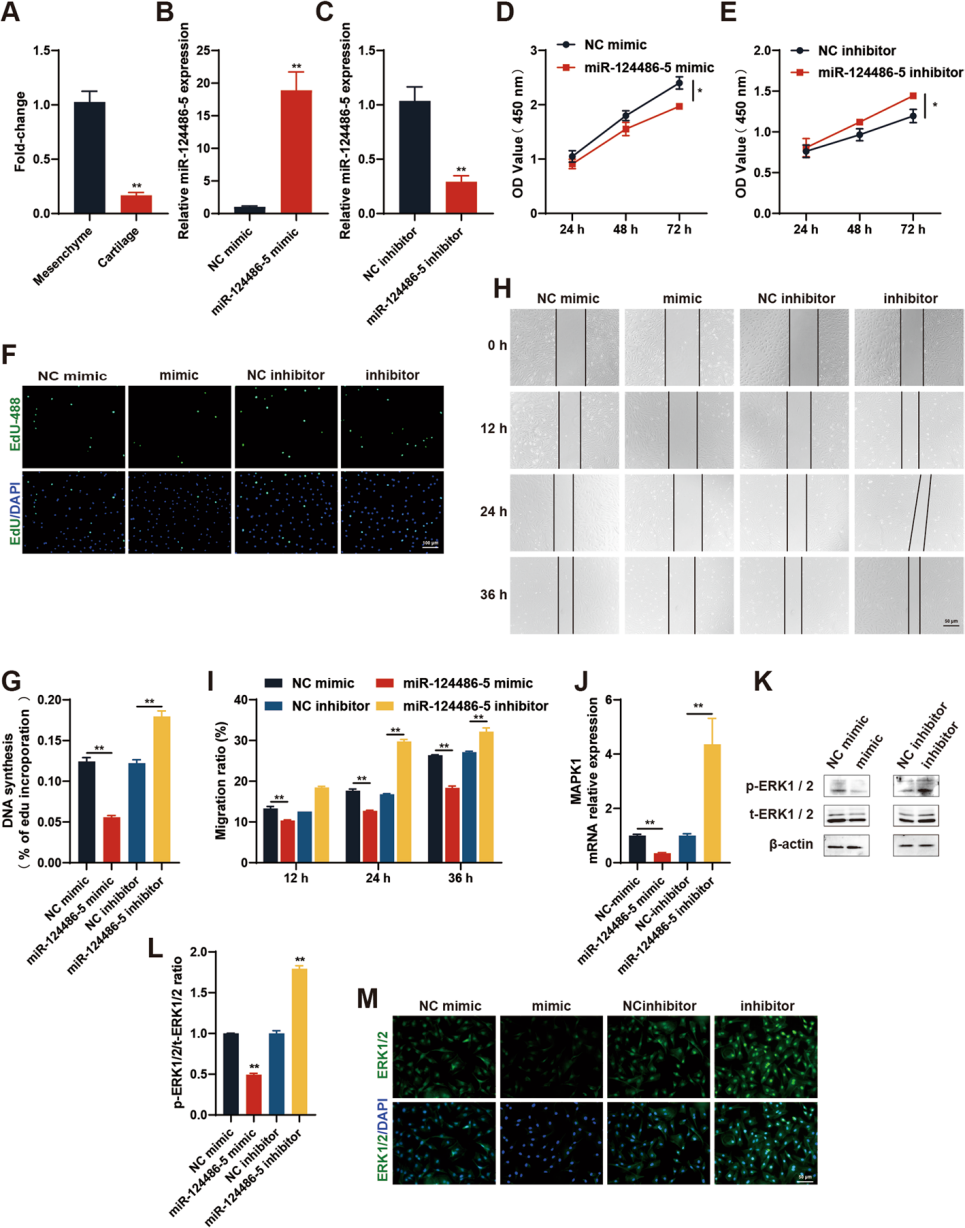
miR-124486-5 inhibits antler chondrocyte proliferation and migration by targeting the 3ʹ UTR of MAPK1. **A** Less miR-124486-5 expression was observed in antler cartilage than in mesenchyme tissue. **B** and **C** Fold changes in miR-124486-5 expression in antler chondrocytes transfected with the miR-124486-5 mimic and inhibitor. **D**–**I** Effects of the miR-124486-5 mimic and inhibitor on antler chondrocytes, determined by CCK-8, EdU, and cell migration assays. Scale bars, 100 (**F**) and 50 (**H**) μm. **J**–**M** qRT-PCR, Western blot, and immunofluorescence analysis of MAPK1 mRNA, t-ERK1/2 and p-ERK1/2 protein expression in antler chondrocytes transfected with the miR-124486-5 mimic or inhibitor. Scale bar, 50 μm. *p < 0.05, **p < 0.01. N.S., no signification

MiR-124486-5 exhibited complementary base pairing with the 3ʹ UTRs of circRNA3634 and MAPK1(Fig. 4D, F). Therefore, we conducted a study to investigate the biological functions of miR-124486-5 in antler chondrocytes. qRT-PCR revealed much less miR-124486-5 expression in antler cartilage tissue than in mesenchyme tissue (Fig. 5A). qRT-PCR performed after the transfection of then miR-124486-5 mimic into antler chondrocytes showed significant overexpression of miR-124486-5 (Fig. 5B). Conversely, an miR-124486-5 inhibitor markedly suppressed miR-124486-5 expression (Fig. 5C). CCK-8, EdU, and cell migration assays demonstrated that the miR-124486-5 mimic inhibited the proliferation and migration of antler chondrocytes, whereas the miR-124486-5 inhibitor promoted them (Fig. 5D–I). qRT-PCR, western blot, and immunofluorescence assays showed that the miR-124486-5 mimic decreased the MAPK1 mRNA, t-ERK1/2 and p-ERK1/2 proteins expression, and the p-ERK1/2 level against the amount of t-ERK1/2 expression, whereas the miR-124486-5 inhibitor increased it (Fig. 5J–M). These findings suggest that miR-124486-5 exerts its function in antler chondrocytes through interaction with MAPK1.

#### CircRNA3634 upregulates MAPK1 by targeting miR-124486-5

**Fig. 6:**
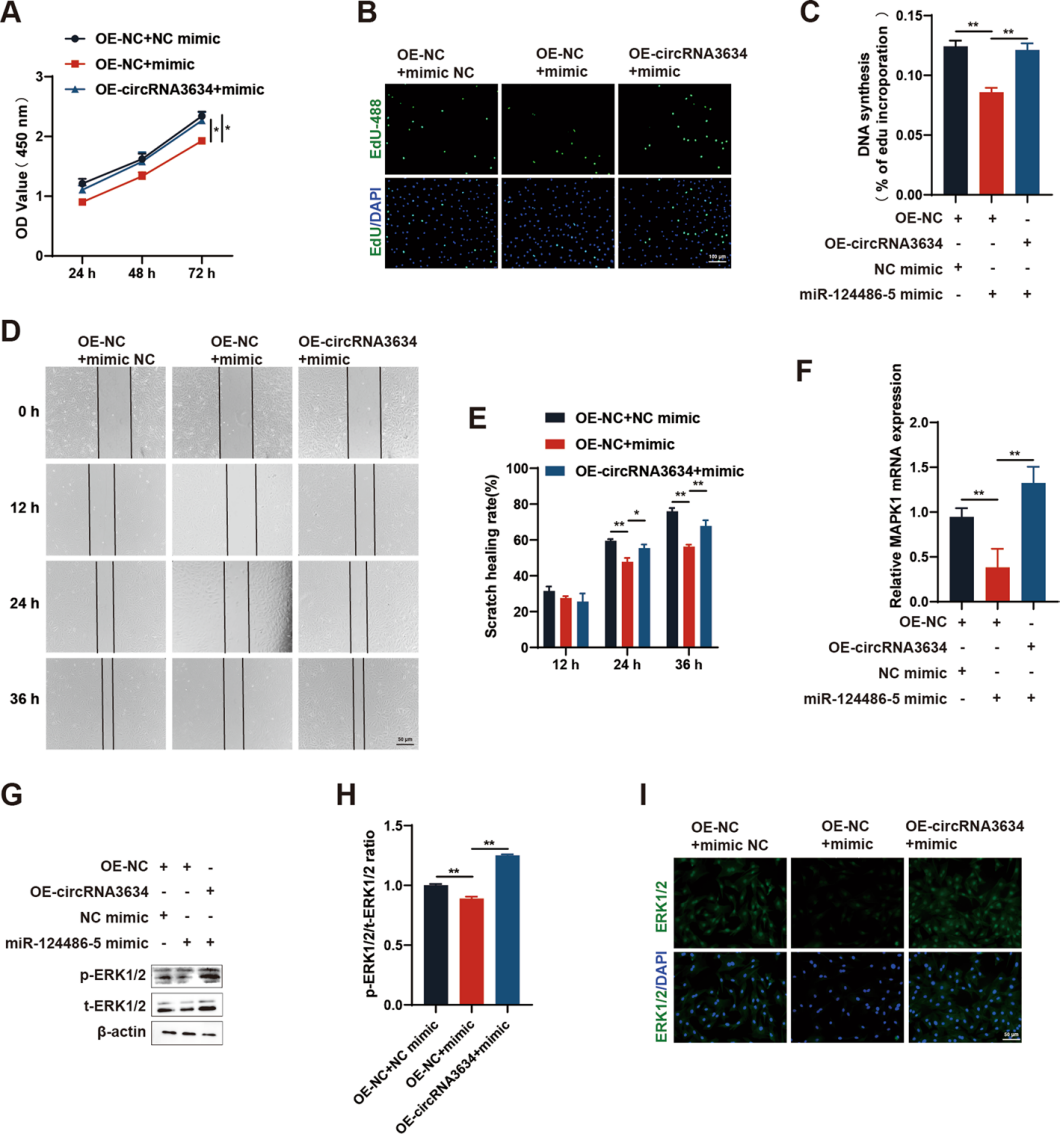
circRNA3634 regulates antler chondrocytes proliferation and migration via miR-124486-5. **A**–**C** Proliferation of antler chondrocytes co-transfected with pcDNA3.1-circRNA3634 and the miR-124486-5 mimic, determined by CCK-8 and EdU assays. Scale bar, 100 μm.** D** and **E** Cell migration assay results for antler chondrocytes co-transfected with pcDNA3.1-circRNA3634 and the miR-124486-5 mimic. Scale bar, 50 μm. **F**–**I** qRT-PCR, western blot, and immunofluorescence results showing that the decreases in MAPK1 mRNA, t-ERK1/2, and p-ERK1/2 protein expression caused by the miR-124486-5 mimic were partially rescued by OE-circRNA3634. Scale bar, 50 μm. *p < 0.05, **p < 0.01. N.S., no signification

Antler chondrocytes were co-transfected with pcDNA3.1-circRNA3634 and the miR-124486-5 mimic to assess whether circRNA3634 counteracted the inhibitory effects of the mimic. CCK-8 and EdU assays showed that OE-circRNA3634 partially restored the miR-124486-5 mimic–induced inhibition of antler chondrocyte proliferation (Fig. 6A–C). A cell migration assay showed that OE-circRNA3634 partially rescued the miR-124486-5 mimic–induced inhibition of antler chondrocyte migration (Fig. 6D, E). qRT-PCR, western blot, and immunofluorescence analyses revealed that the decrease in MAPK1 mRNA expression, t-ERK1/2 and p-ERK1/2 protein expression caused by the miR-124486-5 mimic could be partially reversed by OE-circRNA3634 (Fig. 6F–I). These fundings collectively suggest that circRNA3634 regulates the proliferation and migration of antler chondrocytes through the circRNA3634-miR-124486-5-MAPK1 axis.

#### si-METTL3 reduces circRNA3634 stability and proliferation and differentiation of antler chondrocytes

**Fig. 7:**
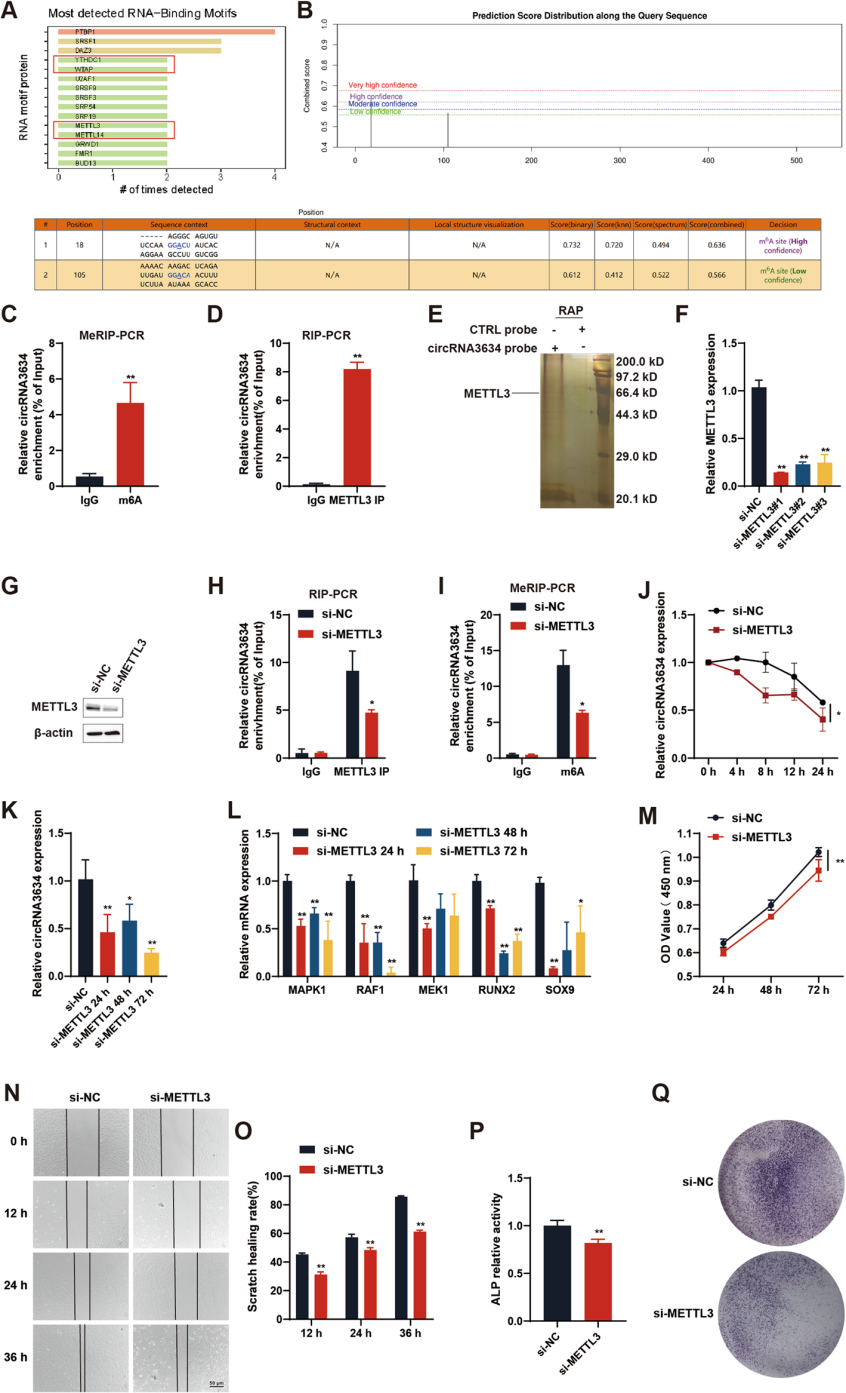
METTL3 reduces the stability of circRNA3634. **A** catRAPID prediction results of RBPs of circRNA3634. **B** SRAMP prediction of potential m6A sites on circRNA3634. **C** and **D** MeRIP-PCR and RIP-PCR demonstrated significant enrichment for circRNA3634 in m6A and METTL3 antibody precipitation complex enrichment. **E** RAP identification of the circRNA3634-protein enriched by circRNA3634 junction probe. **F** qRT-PCR measurement of METTL3 mRNA expression in antler chondrocytes transfected with METTL3 siRNA#1/2/3. **G** Western blot showing METTL3 protein expression in antler chondrocytes transfected with METTL3 siRNA. **H** and **I** RIP-PCR and MeRIP-PCR showed decreased enrichment of circRNA3634 in the m6A antibody precipitation complex after METTL3 siRNA transfection. **J** and **K** Reduced stability and expression of circRNA3634 after METTL3 siRNA transfection, detected by qRT-PCR. **L** qRT-PCR showed decreased expression of MAPK1, RAF1, MEK1, RUNX2, and SOX9 after METTL3 siRNA transfection. **M** CCK-8 analysis of the cell proliferation ability in antler chondrocytes transfected with METTL3 siRNA. Scale bars, 100 μm. **N** and **O** Cell migration assay results for antler chondrocytes transfected with METTL3 siRNA. Scale bar, 50 μm. **P** and **Q** ALP activity assay and ALP staining to detect si-METTL3 effects on antler chondrocyte differentiation. *p < 0.05, **p < 0.01. N.S., no signification

To further explore the potential molecular mechanism by which circRNA3634 promotes the proliferation, migration, and differentiation of antler chondrocytes, we used catRAPID (http://www.tartaglialab.com/) to predictively identify RBPs that interacted with circRNA3634. The prediction results indicated that the RBPs METTL3, METTL14, WATP, and YTHDC1 may interact with circRNA3634, suggesting that circRNA3634 is associated with m6A methylation (Fig. 7A). We used SRAMP (http://www.cuilab.cn/sramp) predict the presence of two potential m6A sites in circRNA3634, with one site located near its splice junction (Fig. 7B). MeRIP-PCR analysis revealed significant enrichment of the exon 20-16 junction region of circRNA3634 in the m6A antibody immunoprecipitation complex, confirming the presence of m6A modification in (Fig. 7C). Among the enzymes involved in m6A methylation, METTL3 serves as the catalytic core [29]. We validated the binding interaction between METTL3 and circRNA3634 through RIP-PCR (Fig. 7D), RAP, and mass spectrometry analysis (Fig. 7E), this interaction was weakened by the reduction of METTL3 expression in antler chondrocytes (Fig. 7F–H). MeRIP-PCR results demonstrated that si-METTL3 led to decreased m6A level of circRNA3634, indicating the influence of METTL3 expression on circRNA3634 m6A modification (Fig. 7I). We also found that si-METTL3 reduced the stability and expression of circRNA3634 (Fig. 7J, K). Meanwhile, si-METTL3 reduced the mRNA expression of MAPK1, RAF1, MEK1, RUNX2, and SOX9 (Fig. 7L). Then, we investigated the effect of si-METTL3 on the proliferation and differentiation of antler chondrocytes. The CCK-8 assays (Fig. 7M) showed that si-METTL3 inhibited the proliferation of antler chondrocytes, and cell scratch assays (Fig. 7N, O) revealed that si-METTL3 inhibited the migration of antler chondrocytes. ALP activity assay and ALP staining showed that si-METTL3 decreased ALP activity and osteogenic differentiation in antler chondrocytes (Fig. 7P, Q).

#### YTHDC1 promotes the nuclear export of circRNA3634 through m6A modification

**Fig. 8:**
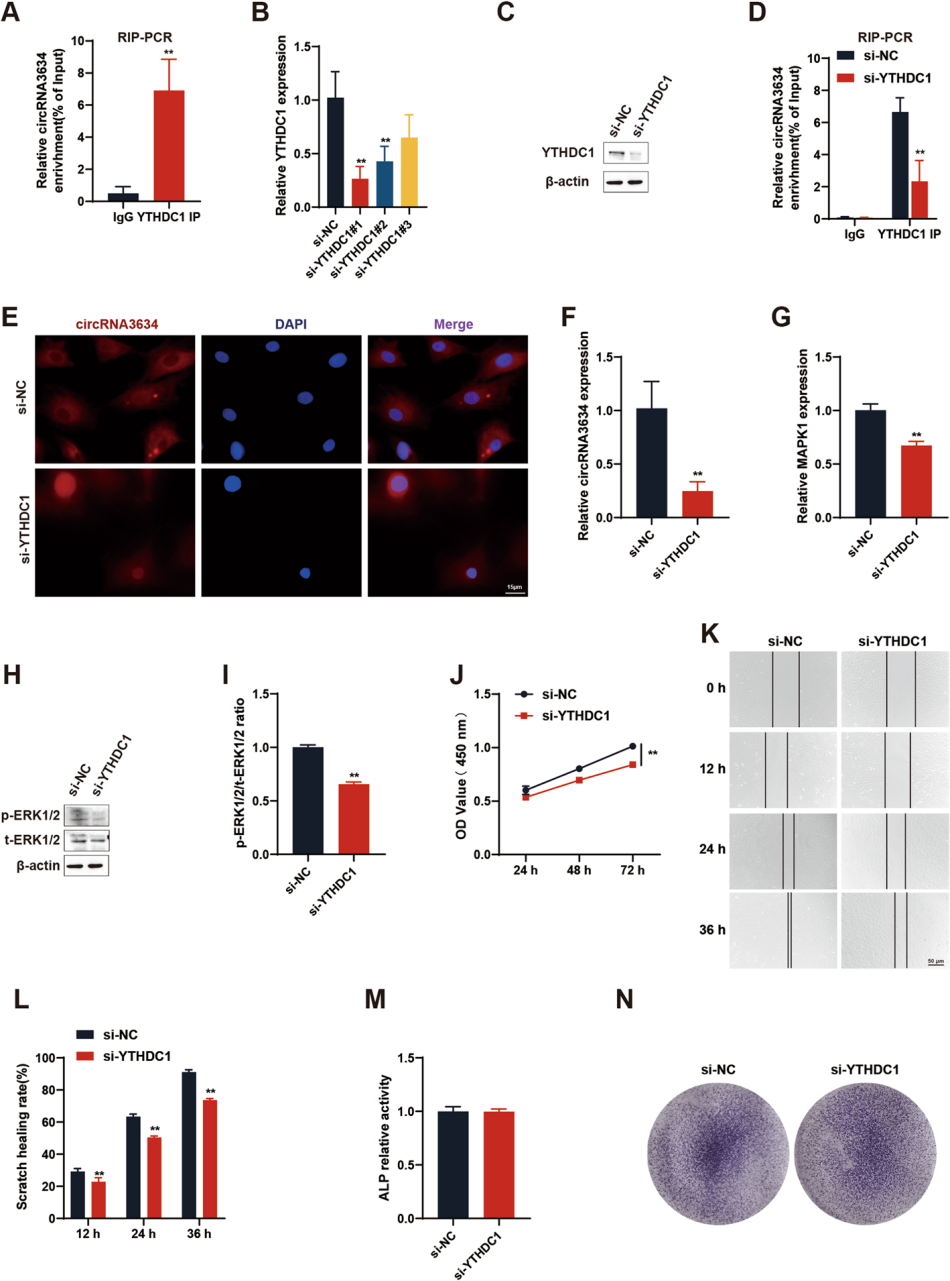
YTHDC1 promotes the nuclear export of m6A-modified circRNA3634. **A** RIP-PCR showed the enrichment of circRNA3634 in the YTHDC1 antibody precipitation complex. **B** qRT-PCR measurement of YTHDC1 mRNA expression in antler chondrocytes transfected with YTHDC1 siRNA#1/2/3. **C** Western blot showed decreased YTHDC1 protein expression in antler chondrocytes transfected with YTHDC1 siRNA. **D** RIP-PCR showed decreased enrichment of circRNA3634 in the YTHDC1 antibody precipitation complex after YTHDC1 siRNA transfection. **E** FISH revealed the predominantly nuclear localization of circRNA3634 upon YTHDC1 siRNA transfection. Scale bar, 15 μm. **F** qRT-PCR showed the reduced expression of circRNA3634 in antler chondrocytes following YTHDC1 siRNA transfection. **G, H** Expression of MAPK1 mRNA and proteins of p-ERK1/2 and t-ERK1/2 in antler chondrocytes transfected with YTHDC1 siRNA was detected by qRT-PCR and western blot.** I** p-ERK1/2 relative ratio. **J** CCK-8 analysis of the cell proliferation ability in antler chondrocytes transfected with YTHDC1 siRNA. Scale bars, 100 μm. **K** and **L** Cell migration assay results for antler chondrocytes transfected with YTHDC1 siRNA. Scale bar, 50 μm. **N**–**O** ALP activity assay and ALP staining to detect si-YTHDC1 effects on antler chondrocyte differentiation. *p < 0.05, **p < 0.01. N.S., no signification

YTHDC1, an m6A reader protein, recognizes m6A sites and promotes the nuclear export of m6A-modified mRNAs and circRNAs [28, 35]. RIP-PCR demonstrated the binding interaction between YTHDC1 and circRNA3634 (Fig. 8A). This interaction was weakened in antler chondrocytes with decreased YTHDC1 expression (Fig. 8B–D). FISH showed that circRNA3634 transfected with YTHDC1 siRNA in antler chondrocytes for 48 h was localized predominantly in the nucleus, rather than the cytoplasm (Fig. 8E). qRT-PCR showed that si-YTHDC1 reduced circRNA3634 expression (Fig. 8F). These findings provide evidence supporting the role of YTHDC1 in binding to circRNA3634 and facilitating its nuclear-to-cytoplasmic export in an m6A-dependent manner. The qRT-PCR and western blot results showed that si-YTHDC1 decreased the MAPK1 mRNA expression and the p-ERK1/2 and t-ERK1/2 protein expression (Fig. 8G–I). After that, we examined whether si-YTHDC1 affected the proliferation and differentiation of antler chondrocytes. The CCK-8 (Fig. 8J) and cell migration assays (Fig. 8K, L) showed that si-YTHDC1 inhibited the proliferation and migration ability of antler chondrocytes. The results of ALP activity assay and ALP staining assays showed that si-YTHDC1 had no significant effect on antler chondrocyte differentiation (Fig. 8M, N).

## Discussion

Antler growth is very rapid, reaching about 2.75 cm/day in late spring and early summer [40]. Researchers have investigated the mechanisms of this rapid growth from various perspectives involving tissue transplantation, the investigation of the antler stem cell microenvironment, multi-omics analysis, and the examination of growth factors, epigenetics, and signaling pathways [7, 8, 41, 42]. Our previous transcriptome sequencing analysis of mesenchyme and cartilage tissues from the *C*. *nippon* antlers revealed that circRNAs and mRNAs are associated with processes such as cartilage development, bone formation, and cell proliferation and differentiation [39]. Based on these findings, we investigated the biological functions of MAPK1 in antler chondrocytes from a circRNA perspective, aiming to provide a theoretical basis for the further understanding of the mechanisms underlying rapid deer antler regeneration and growth.

MAPK1 participates in biological processes such as cell proliferation, differentiation, and survival. In this study, we investigated the involvement of MAPK1 in the regulation of antler chondrocyte proliferation and differentiation. We observed significant upregulation of MAPK1 in antler cartilage tissues, suggesting that MAPK1 plays a role in the regulation of antler growth and development. We determined that the inhibition of MAPK1 expression significantly suppressed antler chondrocyte proliferation, confirming the crucial regulatory role of MAPK1. The ERK signaling pathway plays a dual role, promoting or inhibiting in cartilage formation and chondrocyte differentiation. For instance, ERK MAPK mediates CTGF/hcs24-induced chondrocyte proliferation and p38 MAPK mediates chondrocyte differentiation [43]. The inhibition of ERK1/2 may hinder osteogenic differentiation [44]. Conversely, under the influence of TGF-β1, the ERK signaling pathway inhibitor PD98059 promoted the chondrogenic differentiation of rat bone marrow mesenchymal stem cells and the p38 signaling pathway inhibitor SB20350 inhibited this differentiation [45]. These studies were conducted with different cell types, developmental stages, and culture durations, which may explant the diverse functional roles observed for the ERK pathway. We further found that MAPK1 regulates the differentiation of antler chondrocytes by modulating the expression of RUNX2 and SOX9, expanding our understanding of the role of MAPK1 in the antler chondrocytes’ osteogenic differentiation. The precise regulation of MAPK1 may contribute to offer novel strategies for antler regeneration and the treatment of related diseases.

CircRNAs play crucial roles in processes such as extracellular matrix (ECM) degradation, cartilage differentiation, inflammation, and the development, invasion, and metastasis of various diseases [24, 46–48]. However, the roles and regulatory mechanisms of circRNAs in antler-related processes remain largely unknown. In this study, we focused on circRNA3634, which is highly expressed in antler cartilage tissue and originates from the COG3 pre-mRNA. Like most circRNAs, circRNA3634 maintained its structural integrity when exposed to RNase R. Furthermore, its half-life exceeded that of the linear mRNA of COG3. In in-vitro experiments, circRNA3634 promoted the proliferation and migration of antler chondrocytes. OE-circRNA3634 led to increased mRNA and protein expression of the target gene MAPK1.

Exon-derived circRNAs are commonly localized in the cytoplasm and competitively bound to miRNAs, forming complexes with the AGO2 protein and thereby relieving the miRNA-mediated inhibition of target mRNAs [49]. We performed FISH in this study, which corroborated the cytoplasmic localization of circRNA3634. Through RIP-PCR and dual-luciferase assays, we confirmed the binding interaction between circRNA3634 and miR-124486-5. Functional cell experiments demonstrated that miR-124486-5 exerts a negative regulatory effect on the proliferation, migration, and differentiation of antler chondrocytes by downregulating the expression of MAPK1. Rescue experiments indicated that the inhibitory effects of miR-124486-5 on cell proliferation, migration, and differentiation were partially restored by OE-circRNA3634. These findings strongly support the role of circRNA3634 as a ceRNA influencing the activity of MAPK1 in antler chondrocytes through miR-124486-5. Further investigation to precisely characterize the mechanisms by which circRNA3634 functionally regulates antler chondrocytes will contribute to the identification of uncovering key regulatory factors involved in cartilage development and disease progression. This, in turn, will enhance our understanding of chondrocyte development and functional regulation.

Although m6A modification has been demonstrated to exist widely in mRNAs and ncRNAs, the majority of research has focused on mRNAs. In addition, research on the impact of m6A modification on circRNA biology is limited. OE-METTL3 in H1975 cells accelerated the degradation of SLC7A11 mRNA [50]. Additionally, m6A modification has been shown to regulate the self-renewal and differentiation of mouse embryonic and glioblastoma stem cells by promoting mRNA decay [51, 52]. In HCC cells, si-METTL3 reduced the expression of circRNA-SORE, and si-IGFBP2 decreased the stability of circMDK [53, 54]. m6A modification likely exerts distinct regulatory mechanisms in the modulation of mRNA and circRNA stability and expression. We investigated the impact of m6A modification of circRNA3634 in antler chondrocytes. MeRIP-PCR revealed the presence of m6A modification sites near the splicing site of circRNA3634. The m6A modification of circRNA3634 decreased with lower METTL3 expression, strongly suggesting an association between them. Moreover, the down-regulation of METTL3 in antler chondrocytes led to reduced circRNA3634 stability and expression. These findings are consistent with previous studies suggesting that m6A modification enhances the stability of circRNAs [53, 55]. Further investigation is necessary to uncover the underlying mechanisms at a deeper molecular level. Recent studies have revealed the role of METTL3 in cell proliferation and differentiation. Knockdown of METTL3 increased accumulation of DNA damage, reduces cell viability, in both MCF-7 and MB231 cell [56]. METTL3 has been reported to involved in osteogenic and adipogenic responses in mesenchymal stem cells (MSCs) [57]. Here, we found that si-METTL3 inhibited the proliferation, migration, and differentiation of antler chondrocytes and reduced the expression of ERK signaling pathway-related genes. Our data enrich the evidence that m6A regulates the proliferation and differentiation of antler chondrocytes, and provide new ideas for the study of antler growth and development.

mRNAs transcription occurs in the nucleus and mRNA translation takes place in the cytoplasm. Similarly, circRNAs are generated primarily in the nucleus, but act as miRNA sponges in the cytoplasm [58]. Thus, the process of ceRNA regulation from the nucleus to the cytoplasm is merits exploration. Roundtree et al. [28] found that YTHDC1 mediates the export of methylated mRNAs in HeLa cells, and that YTHDC1 knockdown leads to the accumulation of m6A-modified mRNA in the nucleus. Rong et al. [59] demonstrated that YTHDC1 promotes the cytoplasmic output of m6A-modified circHPS5 in HCC cells. In our study, circRNA3634 was localized primarily in the nucleus, with a small fraction remaining in the cytoplasm of antler chondrocytes after YTHDC1 knockdown. The expression of YTHDC1 in antler chondrocytes was reduced by approximately 75% after transfection with YTHDC1 siRNA for 48 h, several possibilities may be offered to explain this observation. First, the residual YTHDC1 may have continued to participate in the nuclear export of m6A-modified circRNA3634. Second, as circRNA3634 has a half-life > 24 h, FISH may have detected circRNA3634 localized in the cytoplasm before YTHDC1 siRNA transfection that had not degraded. Third, other transport mechanisms that do not undergo m6A methylation may be involved in the nuclear-cytoplasmic export of circRNA3634 in antler chondrocytes.

We found that si-YTHDC1 reduced the expression of circRNA3634. Combined with FISH results, this finding suggests that the nuclear retention of circRNA3634 contributes to the reduction of its expression. A recent study revealed that si-YTHDC1 in HeLa cells leads to the reduction of the expression of circ-ZNF609, accompanied by an increase in its precursor transcript, while the level of the corresponding linear mRNA remains unchanged [29]. These findings suggest that YTHDC1 is involved in the reverse splicing of circ-ZNF609. Interaction between DDX5 and YTHDC1 promotes the splicing of pre-mRNA into mature circRNA in rhabdomyosarcoma cells [60]. Thus, the down-regulation of YTHDC1 expression in antler chondrocytes may weaken the splicing of COG3 pre-mRNA into mature circRNA3634 potentially contributing to the reduction of circRNA3634 expression. Further experimental validation is need to fully elucidate the underlying mechanism. In our results, YTHDC1 was involved in the nuclear export of circRNA in antler chondrocytes and reduced the expression of p-ERK1/2 and t-ERK1/2. In addition, our study found that si-YTHDC1 inhibited the proliferation and migration of antler chondrocytes, but no significant changes in cell differentiation were observed. These indicate that a sophisticated system exists to mediate YTHDC1’s effects on biological processes. More studies are needed to dissect the role of YTHDC1 in antler chondrocytes.

**Fig. 9:**
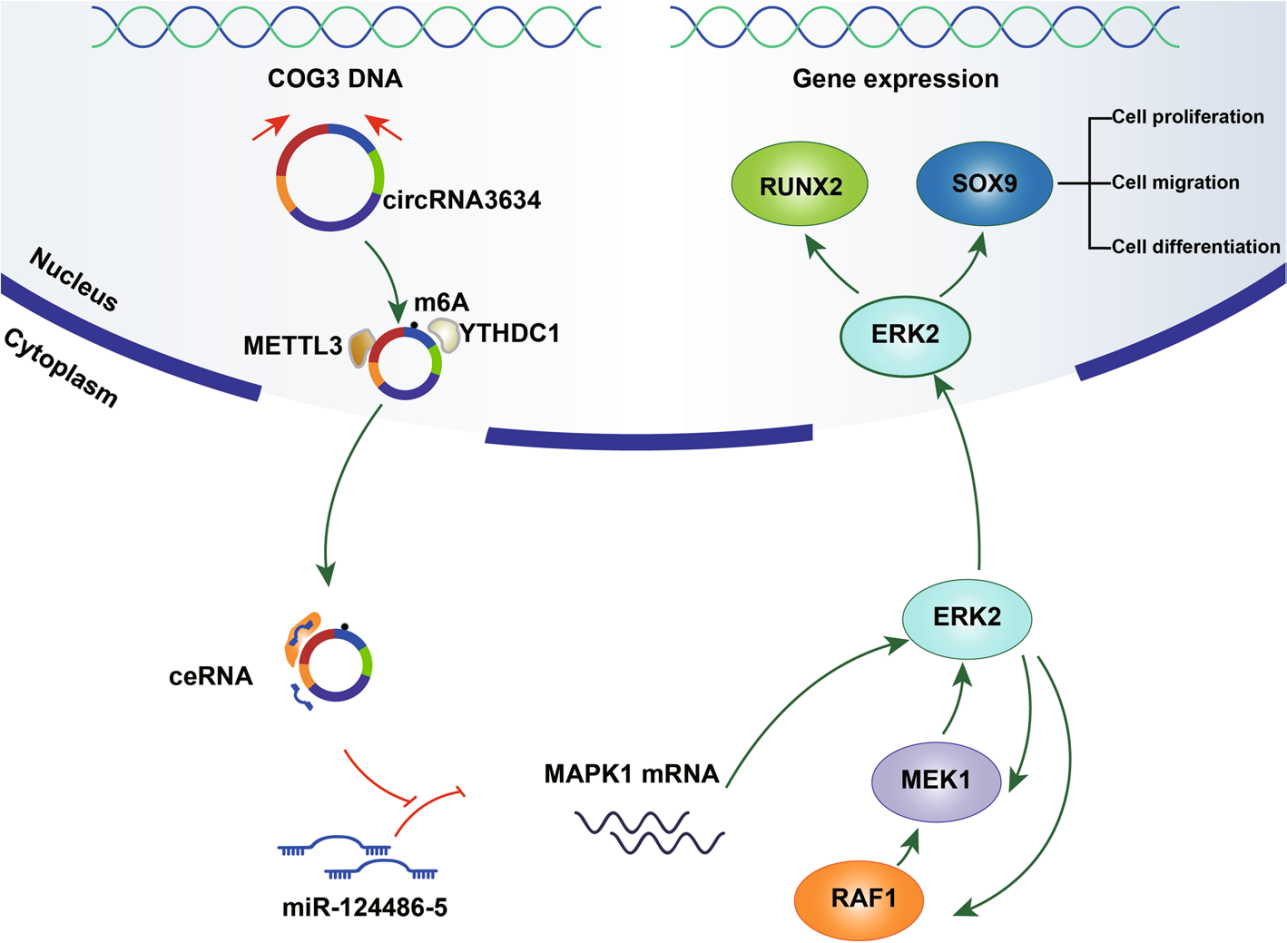
Schematic showing that m6A-modified circRNA3634 promotes the proliferation and differentiation of antler chondrocytes by targeting miR-124486-5—MAPK1. CircRNA3634, originating from COG3 pre-mRNA, undergoes m6A methylation modification facilitated by METTL3 and is subsequently recognized by the reader protein YTHDC1, enabling its export to the cytoplasm. In the cytoplasm, circRNA3634 binds to miR-124486-5, counteracting its suppressive effect on MAPK1. The upregulation of MAPK1 plays a significant role in the promotion of antler chondrocyte proliferation, migration, and differentiation

In summary, our findings demonstrate that MAPK1 is a positive regulator of antler chondrocyte proliferation and differentiation. Additionally, we showed that circRNA3634 is upregulated in antler chondrocytes and functions as a ceRNA to promote these cells’ proliferation, migration, and differentiation through the miR-124486-5-MAPK1 axis. METTL3 is involved in m6A modification of circRNA3634 and proliferative differentiation of antler chondrocytes. Mechanistically, the m6A reader YTHDC1 facilitates the nuclear export of circRNA3634 in an m6A methylation-dependent manner (Fig. 9). Our findings provide the first insight into the involvement of m6A modification in antler chondrocyte processes and a new perspective for the investigation of the mechanisms underlying rapid antler growth.

## Data Availability

The data that support the findings of this study are available from the corresponding author upon reasonable request.

## Abbreviations

MAPK1: Mitogen-activated protein kinase 1
m6A: N6-methyladenosine
OE-circRNA3634: Over expression of circRNA3634
OE-MAPK1: Over expression of MAPK1
si-circRNA3634: Silencing circRNA3634
si-MAPK1: Silencing MAPK1
si-METTL3: Silencing METTL3
si-YTHDC1: Silencing YTHDC1
